# Pinot Blanc: Impact of the Winemaking Variables on the Evolution of the Phenolic, Volatile and Sensory Profiles

**DOI:** 10.3390/foods9040499

**Published:** 2020-04-15

**Authors:** Amanda Dupas de Matos, Edoardo Longo, Danila Chiotti, Ulrich Pedri, Daniela Eisenstecken, Christof Sanoll, Peter Robatscher, Emanuele Boselli

**Affiliations:** 1 Faculty of Science and Technology, Free University of Bozen-Bolzano, Piazza Università 5, 39100 Bolzano, Italy; a.dupasdematos@massey.ac.nz (A.D.d.M.); emanuele.boselli@unibz.it (E.B.); 2 FEAST and Riddet Institute, Massey University, Palmerston North 4410, New Zealand; 3 Oenolab, NOI Techpark, via Alessandro Volta 13, 39100 Bolzano BZ, Italy; 4 Institute for Fruit Growing and Viticulture, Laimburg Research Center, Laimburg 6, I-39051 Pfatten, Italy; danila.chiotti@laimburg.it (D.C.); ulrich.pedri@laimburg.it (U.P.); 5 Institute for Agricultural Chemistry and Food Quality, Laimburg Research Center, Laimburg 6, I-39051 Pfatten, Italy; daniela.eisenstecken@laimburg.it (D.E.); christof.sanoll@laimburg.it (C.S.); peter.robatscher@laimburg.it (P.R.)

**Keywords:** Pinot blanc, white wine, phenolic profile, aroma compounds, trained panel, sensory analysis, prefermentative maceration, cold maceration

## Abstract

The impact of two different winemaking practices on the chemical and sensory complexity of Pinot Blanc wines from South Tyrol (Italy), from grape pressing to the bottled wine stored for nine months, was studied. New chemical markers of Pinot blanc were identified: astilbin and *trans*-caftaric acid differentiated the wines according to the vinification; *S*-glutathionylcaftaric acid correlated with the temporal trends. Fluorescence analysis displayed strong time-evolution and differentiation of the two wines for gallocatechin and epigallocatechin, respectively. After nine months of storage in bottle, the control wine showed higher amounts of most ethyl esters, acetate esters and octanoic acid, whereas higher alcohols characterized instead the wine obtained with prefermentative cold maceration. The sensory panel found notes of apple and tropical fruit in the control wine and attributed a higher overall quality judgement to it, whereas the cold-macerated wine was described by olfactory intensity, spicy and pear attributes.

## 1. Introduction

Pinot blanc cv. is a grape variety firstly described in ampelography in 1868 [1]. This grape variety had been confused with Chardonnay cv. until the origin of Pinot blanc as an independent mutation of Pinot noir was definitively demonstrated [2]. Nowadays, Pinot blanc is one of the most popular grape varieties cultivated in Germany, Austria and France (Alsace). It is also diffused in Argentina, Brazil, Canada, China, New Zealand, South Africa, United States and Uruguay [1]. 

Pinot blanc has climatic requirements compatible to those normally found in the geographical areas of mountain viticulture, similarly to Chardonnay in terms of heat requirements [3,4]; thus, it is one of the most important grapes grown in some mountain areas of Northern Italy, such as South Tyrol [5]. In Italy, Pinot blanc is also used to produce sparkling wines (e.g., Franciacorta appellation wine). 

According to the production regulations of Pinot Blanc South Tyrol DOC [6], this wine must have the following characteristics: straw yellow color with green hints, a pleasant typical aroma, dry and followed by a noticeable acidity in the mouth. In fact, wines obtained from Pinot blanc grapes have been described as greenish-yellow to gold in color, with a relatively sour taste and from light to moderate body. Besides, the aroma has been described using sensory descriptors such as apple, pear, yellow fruits, sometimes mango and even spicy [7,8]. Aging in oak barrels can give these wines notes of apple and almonds with a hint of spices. The flavor of South Tyrolean Pinot Blanc wines was described with apple, pear, citrus, and green notes, occasionally quince and exotic fruits [9]. Also, the aroma of Austrian Pinot Blanc wines has been studied in order to characterize its typical sensory traits; the sensory descriptors evaluated were pear, apple, quince, banana, citrus fruit, apricot, and caramel [10]. A further research highlighted the typicity of Pinot Blanc wine from Austria, stressing out the sensory attribute “pear aroma”. Indeed, different categories for “pear aroma” were described, such as “overripe pear”, “fresh pear”, “cooked/processed pear”, “exotic pear candy”, and “oily waxy pear-like” [11]. 

While Pinot Blanc is considered as a non-aromatic grape variety, it is important to investigate the role of the chemical profiles for authenticity proposes associated with the sensory traits of this wine. According to previous literature, the volatile compounds playing a role in the reported sensory descriptors should be monoterpenes, C_6_ alcohols, aromatic alcohols, and norisoprenoids [7], in addition to alkyl esters [10,11]. Only limited literature is available on the chemical and sensory profiles of Pinot blanc wines [7,9,10,11,12,13,14,15] in comparison to wines obtained from other grape varieties such as Chardonnay. In addition, new information on Pinot blanc winemaking is needed to associate different Pinot blanc wine styles with their main chemical markers and sensory descriptors in the area of South Tyrol. This work aims at incrementing the knowledge on Pinot blanc wine identity in relation to different winemaking practices. The focus is to contribute to the overall assessment of Pinot blanc authenticity and suggest guidelines on the most suitable winemaking practices to obtain a Pinot blanc wine with a notable sensory quality. In one experimental vinification, a prefermentative cold maceration step, the addition of yeast autolysate and the treatment with bentonite after alcoholic fermentation were applied, versus a control vinification, where these steps were not performed. Finally, the relationship between chemical and sensory profiles were discussed.

## 2. Materials and Methods 

### 2.1. Sampling of Grapes

The Pinot blanc grapes were manually harvested in September 2018 in Tirolo (Bozen–Italy, coordinates: 46.693380, 11.144730) in a single and steep vineyard with south-west exposition and 550 m (a.s.l.) altitude. A total of 420 kg of grapes were harvested.

### 2.2. Winemaking Procedures and Sampling

#### 2.2.1. Winemaking

The winemaking process took place at the Experimental Winery of the Laimburg Research Centre, Vadena (Bozen, Italy). Pinot blanc grapes were mechanically destemmed as soon as they were brought to the winery. The whole mass of harvested grapes (420 Kg) was divided in three replicates for each of two different winemaking procedures (70 Kg × 3 repetitions × 2 winemaking procedures, V1 and V2).

The grapes destined for the production of the control wine (V1) were immediately softly pressed in two steps: twice for 10 min at 1 bar and then twice for 10 min at 2 bar. A rest interval of 1 min with press rolling was performed between the two steps in order to avoid grape compacting. Potassium metabisulfite (0.06 g·L^−1^) was added to the must after being placed in 54-L containers. A cold static sedimentation took place at 4 °C overnight completing the preparation of the must before inoculation. The day after, the inoculum was prepared with Zymaflore VL2 yeasts (0.2 g·L^−1^, Laffort Italia S.r.L., Tortona, Italy) followed by the addition of 0.3 g·L^−1^ diammonium phosphate. A second aliquot of diammonium phosphate (0.2 g·L^−1^) was added two days after the inoculum. At the end of the alcoholic fermentation, the wines were racked and cooled down to 4 °C for tartaric stabilization over 12 days.

To produce the experimental wine (V2) the grapes underwent a prefermentative cold maceration in stainless steel tanks at 4 °C for 24 h, with the addition of 6 g h L^−1^ of pectolytic enzyme (Trenolin frio, Erbslöh, Geisenheim, Germany). The same operations used for V1 were applied until the start of the alcoholic fermentation. Unlike V1, the V2 was added with 0.2 g·L^−1^ of a yeast extract (B-energia, HTS Enologia, Marsala, Italy) after six days of fermentation (around the middle of fermentation), as recommended by the yeast extract producer. Also for V2, the wines were racked and cooled down to 4 °C for tartaric stabilization over 12 days at the end of the alcoholic fermentation. Two months after the end of the alcoholic fermentation (and cold stabilization) 0.7 g·L^−1^ of bentonite were added to the V2 wines (Nacalit, Poretec, Erbslöh, Geisenheim, Germany) at 15 °C (cellar temperature). The preparation of the mass of dispersed bentonite lasted one day and the settlement lasted for about 2 weeks. Successively, the wine was racked to proceed with filtration and bottling. All the other steps of the winemaking procedure did not differ from the control wine (V1).

The bottling was performed on the same day for both vinifications (V1 and V2), according to the following procedures: pre-filtration (paperboard filter using firstly 1 μm and then 0.5 μm pore diameter filters) and sterile microfiltration (0.45 μm pore diameter cartridge). No pumps were used for this operation: the wines were poured into a steel tank and later a N_2_ pressure was applied to facilitate the passage of the wines through the filters. To avoid off-flavors, the lines were preliminary rinsed with water and were then pre-conditioned with the wine before filtration. Each filter was replaced at the beginning of each operation. The wines were bottled in 500mL glass bottles and closed with a screw cap. Following the procedure described in the next section, a total number of 78 bottles per wine were obtained (26 bottles × 3 repetitions × 2 winemaking procedures). All bottles were stored at a constant temperature of 16 °C until the opening for the analysis.

#### 2.2.2. Sampling during the Winemaking

The samples were collected at each step of the two vinifications for further analysis and the corresponding flow-charts are shown in Table 1. Each sample was protected from oxidation by pouring them in a 50-mL plastic sealed vial previously filled with N_2_. No empty headspace was left.

### 2.3. Chemical Characterization

#### 2.3.1. Materials

All reagents, analytical standards and solvents used for the analyses were of LC-MS grade.

#### 2.3.2. Determination of Enological Parameters

The samples were monitored using a Foss analyzer (WineScan SO_2_, FOSS Analytical A/S, 69, Slangerupgade, DK 3400, Hilleroed, Denmark). The musts were analyzed for sugars, total acidity, pH, tartaric acid, malic acid, potassium, and yeast available nitrogen (YAN). The wines were analyzed for alcohol content, residual sugars, pH, total acidity, volatile acidity, malic acid, tartaric acid, lactic acid, free and total sulfites.

#### 2.3.3. HPLC-DAD/FLD Profile

The phenolic profile of all the samples was characterized by HPLC analysis. The separation was carried out according to the procedure described by Longo et al. [16], on an ODS column (Eurosphere II, C_18_ stationary phase, 250 × 4.6 mm × 5 µm, Knauer, LabService Analytica, Anzola dell’Emilia, Bologna, Italy) installed on a Nexera X2 UHPLC system (Shimadzu, Milano, Italy) equipped with a UV-Vis diode array detector (DAD, sampling rate 12.5 Hz, time constant = 0.320 s, scan range = 200–800 nm, 1.2 nm slit width) and fluorescence detector (FLD, 10 Hz sampling rate, λ_exc_ = 276 nm, λ_em_ = 316 nm, with 1× gain) in series. The HPLC mobile phase was formed by solvent A (0.1% formic acid in degassed milliQ water) and solvent B (0.1% formic acid in acetonitrile). The gradient method was: 0–2.5 min 1% B, 2.5–50 min 1–25% B, 50–51 min 25–99% B, 51–55 min 99% B, 55–56 min 99–1% B, 56–60 min 1% B. The HPLC flow rate was 0.7 mL·min^−1^. The HPLC peaks were reported as integrated areas vs. retention times using the automatic integration provided by the software (LabSolutions, Shimadzu). The peaks alignment was performed manually. A series of standard compounds were also injected to obtain reference retention times and external calibrations with DAD and FLD (see Table S1 in Supplementary Materials). Peaks of (+)-catechin and (-)-epicatechin were used to correct the 0.3 min retention time shift between DAD and FLD.

#### 2.3.4. Identification of Phenolic Compounds with HPLC-MS

The HPLC previously described was implemented on an Ultimate 3000 UHPLC coupled to a TSQ Quantiva QqQ (Thermo Fisher Scientific, Rodano, Milano, Italy). The MS analyses were run in full-scan and were repeated in three mass ranges: between *m/z* 100-500 (ESI-, monomeric phenols), between *m/z* 500–1200 (ESI+, flavonoids derivatives, proanthocyanidins dimers and trimers) and between *m/z* 1100–1800 (ESI+, proanthocyanidins tetra- to hexamers). Parameters: capillary temperature = 325 °C, capillary voltage 2500 V (neg) and 3500 V (pos), vaporizer temperature = 275 °C, sheath gas: 40 psi, aux gas: 15 psi, sweep gas: 2 psi, full scan 1000 Da/s, Q1 resolution (FWHM) 0.7. Specific tandem-MS/MS experiments were performed on selected species of interest at 25 or 50 eV (collision energy). A series of standards were injected (Table S1) to obtain reference retention times for MS and to correct retention time misalignments with respect to DAD and FLD.

#### 2.3.5. Profile of Volatile Compounds with HS-SPME-GC/MS

A SPME procedure was adapted from a published report [17]. An aliquot (1 mL) of a saturated NaCl solution was placed into a 20 mL glass vial, then 8 mL of wine sample was added. After that, 50 µL of 2-methyl-3-pentanol internal standard (I.S.) solution (dilution 1/50 of I.S. in ethanol, corresponding to a final spiked concentration of 103 mg·L^−1^) was added and the vial was closed with a perforable screw cap, being kept in a heating bath at 40 °C for 5 min with continuous stirring at 300 rpm. Afterwards, stirring was stopped and a SPME fiber (DVB/CAR/PDMS, 50/30 µm, 1 cm) was exposed to the headspace of the 20 mL glass vial for 30 min under continuous heating (40 °C) without stirring, in a configuration similar to the one reported in a published recommended practice [18].

The GC/MS analysis was carried out with manual injection on an Agilent 7890A gas chromatograph coupled to an Agilent 5975 quadrupole mass detector (Agilent Technologies Italia SpA, Cernusco sul Naviglio, Milano, Italy). The thermal desorption took place at 240 °C for 6 min. The separation was performed on a MEGA-WAX Spirit column (0.30 µm/0.18 mm/40 m) in split mode (1:10). Helium carrier gas flow rate was 0.7 mg·L^−1^ (constant flow). The temperature program was: 40 °C for 0.2 min, 40–180 °C at 3 °C min^−1^, then 180–230 °C with 10 °C min^−1^ rate, and finally 3 min at 230 °C. The mass spectrometer operated in EI mode at 70 eV. The mass range was m/z 34–360 at 1 spectrum·s^−1^; the temperature of the ion source and quadrupole were 230 °C and 150 °C, respectively.

The samples were analyzed in a random order to avoid biases. The data integration was performed automatically using the provided tool (Chemstation, Agilent). The total ion current (TIC) peaks were expressed as areas vs. retention time and were manually aligned using the MS spectra to monitor the correctness of the alignment. The peaks were assigned using an integrated approach: (a) by comparison with reference mass spectra (NIST 2011 database); (b) possible isomers/analogues were assigned by calculating the linear retention indexes (LRI) on the base of the elution series of linear alkanes standard (analytical standards C_7_–C_40_ in dichloromethane; Sigma-Aldrich, Milano, Italy), plus additional injection of pure hexane and pentane. Consequently, non-isothermal linear retention indexes (LRI) were calculated for the identified compounds from the reference alkane standards retention times [19]. The NIST library was searched for matching the acquired and reference MS spectra and for comparing measured and theoretical values of LRI for the most likely assignments and the most similar stationary phases.

### 2.4. Sensory Analysis

Eleven subjects (45% females and 65% males, 24 ± 5 years old) were recruited among the students and technical staff at the Free University of Bozen-Bolzano (Bolzano, Italy). A preliminary selection of candidates was carried out in order to choose subjects without any history of oral perception disorders and able to discriminate differences in sensory properties among samples. After a full explanation of the aim of the experiment, an informed consent was signed by suitable candidates who agreed to participate voluntarily in the subsequent training and sensory analysis sessions. The panel received a specific training on how to recognize and evaluate each sensory descriptor using intensity scales (ISO 8586:2012). The training was divided into two phases: qualitative and quantitative evaluations. The initial qualitative analysis phase consisted in presenting two ISO glasses containing two wines (one for each vinification) in order to define the range of descriptors to be used during the following sensory analysis as well as a common sensory vocabulary for visual, olfactory and gustatory evaluations. Following the qualitative analysis, a specific training program developed in sessions of approximately 60 min each (two sessions per week for eight weeks) was undertaken on the descriptors obtained previously. The recognition and classification of aroma standard solutions were performed by asking subjects to identify and recognize different aromas such as rose, orange blossom, elder flower, banana, pear, apple, green apple, lemon, plum, raisin, fig, mint, rosemary, sage, fennel, hay, honey, clove, licorice, anise, and black pepper. In addition, other solutions (alcohol and ethyl acetate) were provided to the panel as ‘pungent’ descriptors. The recognition and classification of standard samples were performed by asking the panelists to identify the taste and to order the standard solutions according to perceived intensity for each descriptor (from the lowest to the highest intensity). The subjects were instructed to use a 9-point intensity horizontal scale ranging from 1 (weak) to 9 (strong) to rate the perceived intensity for taste descriptors including salty (0.25–0.5–0.75–1 g·L^−1^ sodium chloride), sourness (2–4–6–10 g·L^−1^ tartaric acid), sweetness (0.5–2–5–10 g·L^−1^ sucrose), bitterness (0.25–0.5–0.75–1 g·L^−1^ caffeine), and astringency (0.25–0.5–0.75–1 g·L^−1^ alum). All the standard solutions were prepared using food-grade reagents dissolved in a non-aromatic white wine. The subjects were provided with plain crackers without added salt and water and were instructed to rinse their mouth between samples during the sensory sessions.

Following completion of the training and before starting the session, panelists received by the panel leader detailed instructions on the definition of descriptors and how to conduct the sensory evaluation (sampling from the left to the right direction according to the order proposed). They were informed that the descriptors would have been related to visual evaluation (clarity and color intensity), olfactory evaluation (olfactory intensity, floral, apple, pear, tropical fruit, dried fruit, spicy, fresh vegetative, cleanness, off-odor), gustatory evaluation (warmness, sweetness, sourness, saltness, bitterness, astringency), and overall quality judgement. The complete list of descriptors is reported in the Supplementary Material (Table S2). The panel performance was assessed to check the consistency of each panelist and between panelists (data not shown), enabling the identification of assessors who were not consistent with the whole panel, therefore, his/her data was not included in the evaluation.

The wines samples were evaluated according to Quantitative Descriptive Analysis (QDA^®^), which is one of the main descriptive analysis techniques adopted for sensory evaluation, under the conditions described in the UNI 10957:2003 procedure. The wine bottles were opened just before the analysis and 30 mL of wine per glass at around 16 °C were offered randomly (in triplicate) to the panelists in ISO glasses codified with a 3-digit number. The presentation order of the wine samples was randomized between and within participants.

### 2.5. Statistical Analysis

The statistical elaboration of the data was performed using XLStat (version 2019.2.2.59417, Addinsoft, Paris, France) and R (CAT—Chemometric Agile Tool [20]). The phenolic profiles (DAD and FLD) and the volatile profiles (GC-MS) were elaborated with Principal Component Analysis (peak areas non-averaged within the triplicates; variables were mean-centered) using non-scaled (for DAD, FLD and sensory data) or scaled variables (for GC-MS). A selection of chemical (phenolic and volatile compounds; oenological parameters) and sensory variables from PCA was further analyzed by univariate two-way ANOVA (for differences in time and between vinification and their interactions). Significant differences were determined with an α = 0.05 (confidence of 95%) unless stated otherwise.

## 3. Results

The impact of two different winemaking protocols for Pinot blanc were evaluated on the enological parameters, phenolic and volatile profiles from musts to bottled wines. For each vinification, three experimental replicates were studied in parallel. All other factors (e.g., storage of the 54-L glass containers, temperature) were strictly controlled and identical for all samples.

### 3.1. Oenological Parameters

The enological parameters analyzed are reported in Table S3. For the musts, the analyses were performed before the yeast inoculation step. The two musts (T4.V1 and T4.V2) did not show noticeable differences in either sugars content or pH. A slight difference was found in the content of potassium, tartaric acid and malic acid, as V1 showed higher values than V2. The differences in the concentration of potassium could be explained according to the effect of the prefermentative cold maceration, as the maceration could cause higher potassium bitartrate extraction, which in turn can evolve in instability and a higher precipitation rate, also promoted by the lower temperature.

Even if the initial concentration of sugar was not significantly different, after a storage of three months in bottle the wines developed slightly different levels of ethanol (% v/v), 14.9 (W3.V1) and 14.5 (W3.V2) respectively. Consequently, the V2 wine showed higher residual sugars (Table S3); however, the different alcohol contents in the two wines cannot be explained only by the differences in the relative amounts of non-fermented sugars. This could be accounted instead to a slight yeast stress, probably due to a higher initial content of polyphenols in V2 (427 mg·L^−1^) than V1 (388 mg·L^−1^), released by the prefermentative cold maceration [21]. The pH did not show significant differences among the two wines after three months (W3) and six months (W6) in bottle, although the total acidity was slightly higher in V2. Volatile acidity was lower in the experimental wine (V2), likely due to a lower microbial activity. An overall decrease in the level of L-malic acid and the parallel increase of L-lactic acid was also observed in V2, due to malolactic fermentation.

### 3.2. Phenolic Compounds

**Figure 1:**
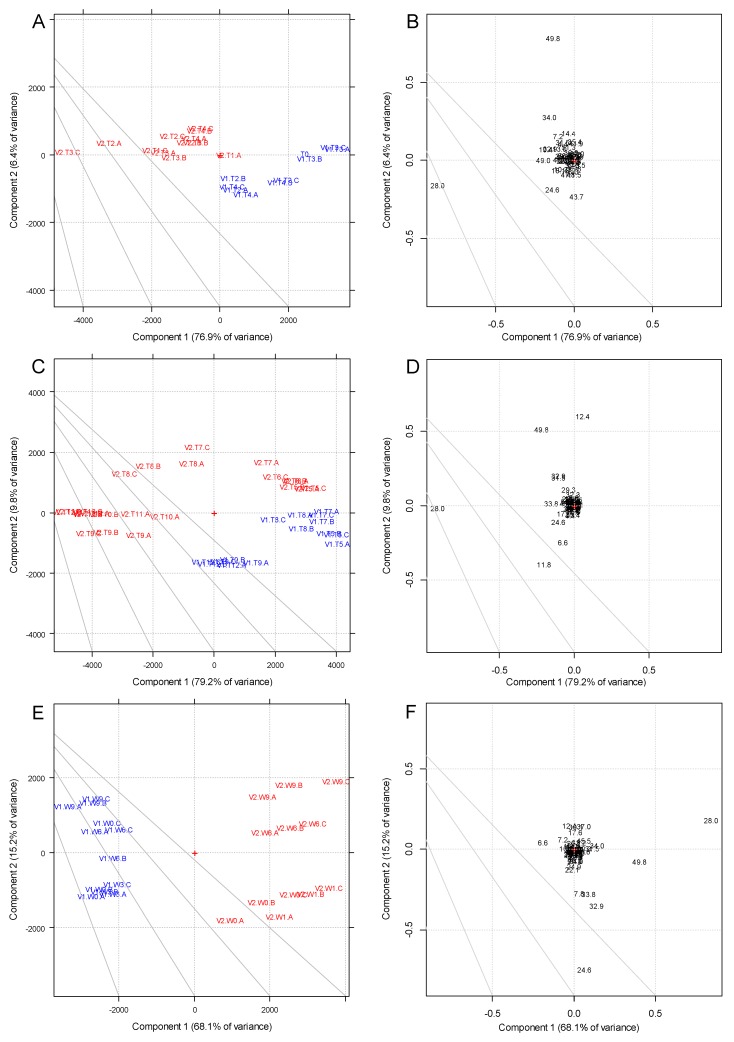
PCA of HPLC-DAD peaks. (**A**) PC1 vs. PC2 score plot for must samples; (**B**) PC1 vs. PC2 loading plot for must samples; (**C**) PC1 vs. PC2 score plot for fermentation and stabilization samples; (**D**) PC1 vs. PC2 loading plot for fermentation and stabilization samples; (**E**) PC1 vs. PC2 score plot for bottled samples; (**F**) PC1 vs. PC2 loading plot for bottled samples. Loadings are indicated with their HPLC retention times as labels. Blue font indicates V1 samples; red font indicates V2 samples. (**A**–**C**) into the PCA plot indicates the three replicates.

Phenolic compounds are very important flavor-active components of wine and they were fully characterized by HPLC-DAD. The absolute concentration of the analytes is not needed to perform multivariate statistical processing, thus the peak areas were treated as previously described in the Section 2.5. Principal Component Analysis (PCA) was performed on the resulting dataset in order to highlight the trends associated to the winemaking processes and time evolution. The PCA plots describing the phenolic compounds are shown in Figure 1 (the score plots are shown in Figure 1A,C,E and the loading plots are in Figure 1B,D,F).

These exploratory PCA plots were obtained by re-grouping the samples in three main datasets: unfermented musts (scores in 1A and loadings in 1B), samples during the alcoholic fermentation and further stabilization (scores in 1C and loadings in 1D), and bottled wines (scores in 1E and loadings in 1F). This choice was due to two main reasons: (i) the overall variance in the dataset of the must samples (T0–T4) was much smaller than the one of the samples during/after fermentation (T5–T12); this lead to the separation between the clusters corresponding to V1 and V2 for T0-T4 (musts) which was discussed when analyzing the T0–T12 samples altogether, and (ii) the PCA performed on the samples after bottling allowed to evaluate directly the evolution of the wine over storage.

A list of all the phenolic compounds identified (these last ones selected according to the PCA results and identified by further LC-MS/MS analysis or standard injections) is reported in Table 2. An example of a chromatogram is reported in Figure S1 left.

In the PCA, two main trends corresponded to clear experimental factors as observed in the score plots in Figure 1A,C,E: vinification and time evolution. Beside a clear separation in observations caused by vinification (“blue” samples V1 vs. “red” samples V2) usually highly correlated to PC1, considering each vinification, a progressive trend in operation (time evolution) can be seen in all three score plots, albeit with different orientations with respect to the main separation along PC1. Whereas the score plots gave a distinctive overview of these trends (e.g., the separation in vinification clearly unfolds along PC1 and it highlighted here by choosing different colors for the sample labels), the loading plots showed which variables were most affected by these trends. In fact, in all three loading plots (Figure 1B,D,F), *trans*-caftaric acid (R.t. 28.0 min) was strongly correlated to PC1 with the effect of vinification. Conversely, a strong temporal trend could be observed clearly aligned to PC2 in Figure 1E and 1C, respectively. Astilbin (taxifolin-3-O-rhamnoside) (R.t. 49.8 min) showed a strong correlation along PC2 for samples T5–T12 (Figure 1D). Astilbin is a flavanonol which was previously observed in French varietal red wines in the presence of noble rot development, along with stilbene oligomers [22]. Taxifolin, too, was tentatively identified in the bottled wines (R.t. 50.8 min, see Table 2).

For must samples (T0–T4) in Figure 1A, a clear separation between V1 and V2 was observed along PC1; this could be associated only to the effect of the pre-fermentative (cold) maceration of the V2 samples. Two variables contributed mostly to PC1 (separation in vinification) and PC2: *trans*-caftaric acid (R.t. 28 min) and astilbin (R.t. 49.8 min). Differently than later samples, no clear temporal trend was however observed along the second principal component.

For fermentation/stabilization samples (T5–T12), both the separation by vinification and the time evolution could be observed. V2 samples at the middle/end of fermentation (T6 and T7) were however closer to the V1 samples than the other ones (Figure 1C); the temporal evolution was also observed, proceeding both for V1 and V2 as a trend from the upper-right quadrant to the lower-left quadrant in the Score plot (Figure 1C). This trend was correlated to the flavanonol astilbin (R.t. 49.8 min) and to a tentatively assigned hexoside derivative of gallic acid (R.t. 12.4 min, λ_MAX_ = 267 nm; mass = m/z 331, ESI -). As with musts (T0–T4, Figure 1A,B), the separation along PC1 in Figure 1C (vinification factor) was strongly correlated with *trans*-caftaric acid. In bottled wines (Figure 1E), both the trends were clearly visible; they were perfectly aligned with PC1 and PC2. However, the temporal trend (PC2) did affect in this case more the samples V2 than V1.

This trend proceeded from lower to higher values of PC2 and it was strongly correlated to GRP (Figure 1F), which decreased from W0 to W9. The complete trends for astilbin, taxifolin, *trans*-caftaric acid and *S*-glutathionylcaftaric acid (grape reaction product or GRP) from T0 to W9 (T16) were displayed in Figure S2. The concentration of GRP in the samples appeared gradually to increase up to W3 and then sharply decreased (see Figure S2), whereas its precursor (*trans*-caftaric acid, R.t. 28.0 min) displayed overall increasing trend with a temporary decrease between T3 and T5 for V2 (start of the fermentation). Since the relative amounts of these two compounds are related over winemaking and storage [23,24], the observed reciprocal trends as well as the sharp decrease for GRP after T14 (W3) were unexpected.

In order to test if the observed differences for these compounds in bottled wines were significant, a two-way ANOVA was performed applying vinification and time as factors (Table S4). GRP, astilbin and taxifolin were significantly affected by the temporal evolution. No effect due to time was observed instead with *trans*-caftaric acid. All compounds were instead significantly affected by vinification, although to a much smaller extent for GRP. These results supported the observations seen in PCA (Figure 1E,F). Interestingly, only taxifolin displayed a minimal significant interaction between the time and vinification factors.

Besides, a PCA analysis of the HPLC-FLD peak table was performed. HPLC-FLD data were recorded in series to the DAD. FLD was set to target monomeric and oligomeric flavan-3-ols (see the Experimental Section). The PCA is reported in the Supplementary Material (Figure S3). The separation between the two vinifications was very clear (PC1). Again, a temporal trend could also be observed (PC2) for bottled wines (SI 3E). Particularly for bottled wines, two of the compounds with the highest absolute loading in PC1 (vinification factor) and PC2 (time factor) were (-)-epigallocatechin (R.t. 31.4 min, higher in V2 than V1) and (-)-gallocatechin (R.t. 24.0 min, decreasing in time), respectively.

### 3.3. Volatile Compounds

**Figure 2:**
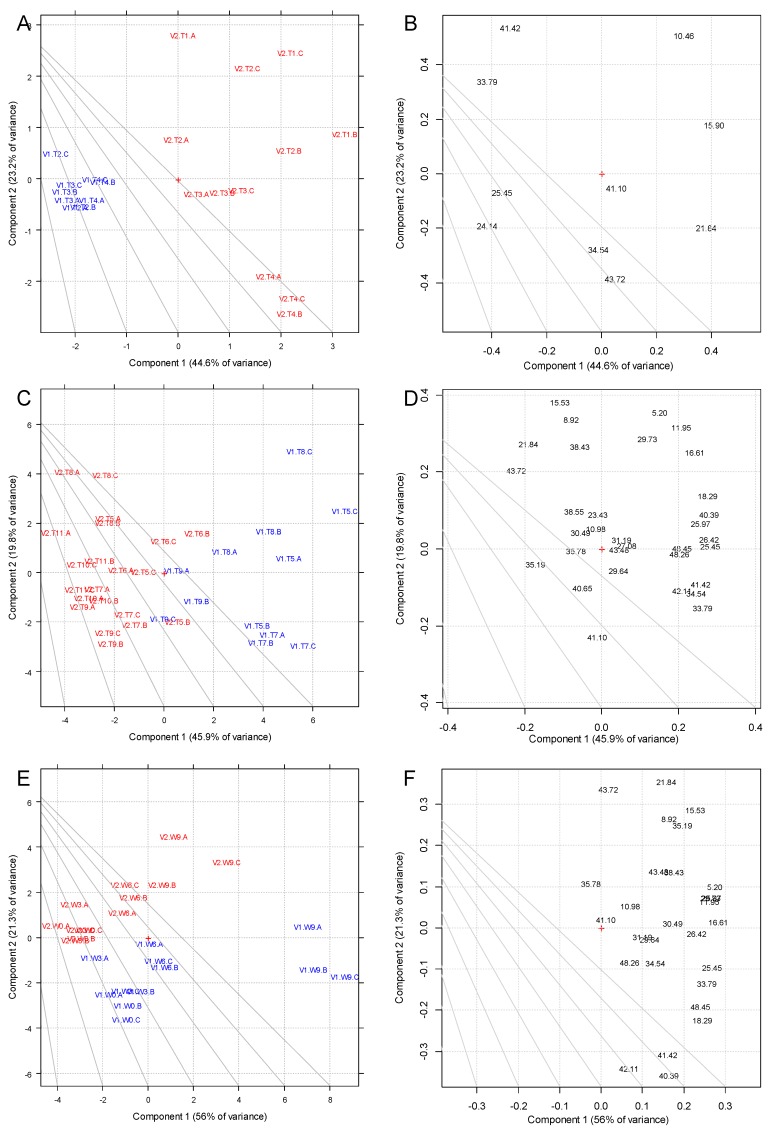
PCA of GC/MS peaks. (**A**) PC1 vs. PC2 score plot for must samples; (**B**) PC1 vs. PC2 loading plot for must samples; (**C**) PC1 vs. PC2 score plot for fermentation and stabilization samples; (**D**) PC1 vs. PC2 loading plot for fermentation and stabilization samples; (**E**) PC1 vs. PC2 score plot for bottled samples; (**F)** PC1 vs. PC2 loading plot for bottled samples. Loadings are indicated with their retention times as labels. Blue font indicates V1 samples; red font indicates V2 samples. A, B, C into the PCA plot indicates the three replicates.

The list of identified volatile compounds identified by HS-SPME-GC/MS (together with the claimed sensory descriptors) was reported in Table 3. The choice of the triphasic phase DVB/CAR/PDMS for SPME was due to the efficient methods reported for wines [25], and applied also for rapid methods followed by Principal Component Analysis of the results [26]. In the Supporting Information (Figure S4) two chromatograms are reported. The analysis of the results was performed using PCA with auto-scaled variables (Figure 2).

The goal of the SPME-GC/MS analysis was not to obtain the absolute concentrations of the volatile compounds present in the wine headspace, but to study the relationships between their relative concentrations (profiles) and the winemaking practices with PCA (Section 2.5). Thus, the internal standard was added to the samples (I.S. in Figure S4) mainly to comply with the original protocol used for this analysis [17] but was not used for the determination of the absolute concentrations of the volatile compounds.

As with the phenolic compounds, the volatiles dataset was split in three parts (musts—Figure 2A,B; fermentation/stabilization samples—Figure 2C,D; wines after bottling—Figure 2E,F), in order to better highlight the trends and to improve the homogeneity among the observations. The PCA of volatile profiles displayed an even higher alignment of the vinification and time evolution factors with PC1 and PC2 respectively, with the noticeable exception of the wines after bottling (Figure 2E,F). Again, score plots and loading plots gave a useful overview of the most relevant trends and the relative affected variables, respectively.

Regarding the influence of V1 and V2 vinifications on the musts (Figure 2A,B), the separation was observed along PC1. At this stage, the operation differentiating the wines was the pre-fermentative cold maceration (only for V2 wines). The variables *n*-hexanol, 2-(*E*)-hexenal and hexanal characterized V2, while the linear ethyl esters and the 2-hexen-1-ol characterized V1. In addition, *n*-hexanol, 2-(*E*)-hexenal, hexanal and 2-hexen-1-ol followed a temporal trend, which was observed along PC2 and characterized particularly V2. This trend must be related to the effects of the pre-fermentative cold maceration (as all other operations so far were the same for the two vinifications). The change along PC2, which mainly involved V2, was then associated to a decrease over time of hexanal (R.t. 10.46 min) and 2-hexenal (R.t. 15.90 min), with an increase of their correspondent alcohols: *n*-hexanol (R.t. 21.84 min) and 2-hexen-1-ol (R.t. 24.14 min), respectively.

The occurrence of C_6_ aldehydes and the corresponding alcohols in wines was related to “leafy/grassy” and “herbaceous” aromas, typical from fresh-cut vegetal tissues [55]. These compounds are originated enzymatically by the activity of lipoxygenases through the oxidation of linoleic and linolenic acids present in the cell wall membranes of the grape skin before fermentation [21,56]. Levels of C*6* aldehydes and alcohols in grapes depend upon the grape variety, the ripeness stage of grapes, treatment of the must, and time and temperature by which the must is kept in contact with the skin, which is the main difference between V1 and V2. During the vinification, these compounds were shown to change due to the chemical reduction of the aldehydes into their corresponding alcohols (which have a higher perception threshold) by the alcohol dehydrogenase of the yeast and to the esterification of the alcohols undertaken by the yeast esterase [57]. This appears to have impacted particularly V2 samples.

The samples collected between the beginning of the fermentation and the bottling (T5–T12) were evaluated with a PCA model as well (Figure 2C,D), which was built separately from the previous samples (T1–T4), for the reason reported above for phenolic compounds. As seen earlier, the variability rapidly increased after T7 and no trend across the initial samples (T1–T4) could be observed, if analyzed together with T5–T12 samples (data not shown). The PC1 vs. PC2 scores and loadings for the samples collected after the beginning of the fermentation are reported in Figure 2C,D. The score plot showed again a separation between V1 and V2. Interestingly, the separation was obtained again along PC1, indicating that the vinification was the main factor influencing the samples variability, with a smaller effect of the temporal trend observed within each vinification, parallel to PC1 this time, indicating that the main variables affected by the different vinification were also causing the evolution of the wines overtime. In fact, the loading plot in Figure 2D shows that the separation in PC1 is mainly caused by the linear ethyl esters and the acetate esters, contributing mostly in V1, and higher alcohols contributing mostly for V2. Noticeably, trends in the T5–T12 PCA Score Plot (Figure 2C) were observed in relation to some classes of volatile compounds. Most branched esters, carboxylic acids (octanoic acid and acetic acid) acetate esters and the linear ethyl esters (with the exception of ethyl butanoate, R.t. 8.92 min) contributed mainly to V1 in PC1. In addition, several groups of volatile compounds associated to a chemical class were observed, even if not specifically associated to one factor or trend. All alcohols were identified at high positive values of PC2 with a negligible but still higher contribution in V2 wines than in V1 (phenylethyl alcohol and n-hexanol, R.t. 43.72 and 21.84 min, respectively).

Finally, the results of the PCA performed on the V1 and V2 samples at W0 (at bottling), W3 (three months after bottling), W6 (six months after bottling) and W9 (nine months after bottling) are presented in Figure 2E,F.

Differently from what was seen with the previous samples, here the wider separation among the observations (Figure 2E) along PC1 was driven by the separation of V1.W9 samples from all the others. A separation between V1 and V2 samples was still present but not clearly defined along one specific PC. Consequently, all samples V1 and V2 were found at lower scores in PC1 than V1.W9. V1.W9 was separated from both V1.W6 and V2.W9 along PC1. Notably, most of the variables were now drawn towards high positive values of PC1, indicating that the volatiles evolution was more accelerated after nine months in V1 than in V2. Perpendicular to the direction descripted by the separation in vinification (V1 vs. V2), a clear temporal trend could also be seen, only now compressed for the samples up to the six-month by the evolution of V1.W9. The two main separations (V1.W9 vs. all other samples and the temporal trend) overlapped, as V1.W9 separation was strongly driven by the temporal evolution. Observing the corresponding loading plot (Figure 2F), such separation was caused mainly by the linear ethyl esters (e.g., ethyl hexanoate, ethyl octanoate and ethyl decanoate, R.t. 16.61, 25.45 and 33.79 min, respectively) and acetates (ethyl acetate and isoamyl acetate, R.t. 5.20 and 11.95 min, respectively). Besides, also isoamyl hexanoate (R.t. 26.42 min), hexyl acetate (R.t. 18.29 min), acetic acid (R.t. 25.97 min) and octanoic acid (R.t. 48.45 min) contributed to the separation of V1.W9 from the other samples. Regarding the general separation of V1 vs. V2, isoamyl decanoate (R.t. 42.11 min), ethyl dodecanoate (R.t. 41.42 min) and phenylethyl acetate (R.t. 40.39) showed a remarkable contribution. Regarding the temporal trend (pointing towards positive values of PC1 and PC2), the most contributing compounds were mainly alcohols, such as phenylethyl alcohol (R.t. 43.72 min), n-hexanol (R.t. 21.84 min), isoamyl alcohol (R.t. 15.53 min) and also ethyl acetate (R.t. 5.20 min). However, as the V1.W9 replicates were so much clustered away from all other samples, the temporal evolution (from W0 to W9) of both V1 and V2 was not so much distinguished (in terms of the PCA score plot) from the separation between the two different vinifications (V1.W6 is closer to V2.W9 than to V1.W9).

In fact, a clear distinction of these two trends (e.g., along a specific direction in the scores plot) in terms of scores and loadings could not be clearly defined, as it was in previous cases (e.g., musts).

Indeed, differences in the aroma profile of the wines could have a substantial impact on its sensory profile. Hence, in order to test the significance of the variables with respect to time and vinification as well as the interaction of these two factors, a two-way ANOVA was applied on all variables used in the PCA (Table S5). The ANOVA showed that most of the variables now affected the temporal evolution significantly (Table S5.B) with a general increasing trend towards V1. However, from the PCA this can be easily imputed solely to V1.W9, which was driving the separation both in vinification and time. However, ANOVA indicated that a significant interaction between time and vinification was present only for some of the volatile compounds such as ethyl acetate, ethyl butanoate, ethyl hexanoate, n-hexanol, acetic acid, isoamyl hexanoate, ethyl nonanoate and 2,3-butanediol.

### 3.4. Sensory Analysis

**Figure 3:**
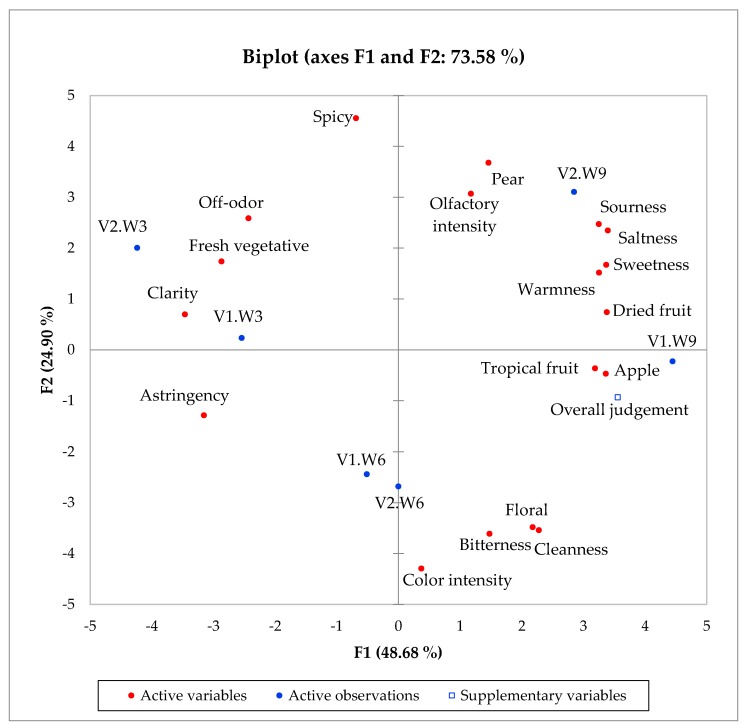
Principal component analysis (PCA) on sensory profile data of Pinot Blanc wines. V: vinification type (1: control wine, 2: wine variant), W: time over storage (months).

PCA performed on all the sensory data (Figure 3) showed that two principal components accounted for 74% of the total variance. PC1 explained well the time—evolution (49% of the variance), whereas PC2 (25% of the variance) seemed only partially to represent the separation by vinification, although its interpretation is less clear.

In this PCA biplot, trends characterizing the scores and the effects on the variables (loadings) are shown. The descriptors that mostly contributed to W3 (at negative values of PC1), hence to the temporal trend by decreasing in time, were clarity, fresh vegetative aroma, off-odor and astringency. On the other hand, color intensity, floral aroma, cleanness, and bitterness described mostly W6 (negative values of PC2). Regarding W9, the sensory descriptors were sourness, saltness, sweetness, warmness, dried fruit, tropical fruit and apple aromas; thus, these variables contributed also substantially to the temporal trend (increasing over time from W3 to W9). Regarding the difference between the two vinifications (V1 vs. V2) at W9 (after nine-month storage), the most relevant sensory descriptors characterizing V1 were tropical fruit aroma, apple aroma, and overall quality judgement. Instead, the most important sensory descriptors that characterized V2 (positive values of PC2) were olfactory intensity, spicy and pear aromas. Taking a look at the PCA considering each wine individually, it is possible to highlight that the wine V1 after nine months of storage (V1.W9) was evaluated with a high overall quality judgement and was described predominantly by the sensory descriptors tropical fruit and apple aromas as well as the absence of off-odor.

To better investigate these trends, a two-way ANOVA was applied on all the sensory variables used in the PCA biplot (Table S6). The aim was to assess the main effect of each independent variable (time and vinification) but also if there was any interaction between them (time × vinification). The ANOVA showed that floral, tropical fruit and cleanness were statistically different for the vinification factor. Considering the factor time, the sensory descriptors different were floral, tropical fruit, dried fruit and fresh vegetative aromas, warmness, sweetness, sourness, saltness, and overall judgement. Finally, considering the interaction, only floral aroma was found significative. This showed an interesting relation with the results shown in Figure 2 for the volatile compounds analyzed by GC-MS: V1 appeared to be the wine with a faster evolution into the bottle, indicated by the separation it showed from previous months and from V2.W9; in fact, V2 evolved as well as from W6 to W9 but more slowly than V1. However, according to the sensory profile, the two wines at W9 are characterized by rather different descriptors, a possible effect of the different operations performed over the vinification styles. In addition, further studies are necessary using other set of Pinot blanc wines under the same vinification conditions to firmly confirm the differences found in this study.

## 4. Discussion

The application of different winemaking practices caused major differences in the chemical, phenolic, volatile and sensory profiles of the final wines investigated. Previous studies showed that maceration of the crushed and destemmed grapes before fermentation led to a higher abundance of phenolic compounds [58]. Although the mild conditions (soft pressing, cold temperature and brief time for the maceration) applied here still allowed for more phenolic compounds to be extracted, the same mild conditions prevented other relevant consequences, such as sharp increases of pH in the macerated samples. The level of specific grape phenolic compounds was seen to be positively affected by maceration, as previously reported [59], in this study particularly for *trans*-caftaric acid and astilbin. Specific effects due to maceration in white grape musts were previously described [60]. However, the specific effect on astilbin was yet unknown, to the best of our knowledge. Besides, an interesting relationship between *trans*-caftaric acid and GRP was observed, which was not expected given previous literature [23], and this indicates that further phenolic evolution occurs during the storage of Pinot blanc, as reported for other white wines [24].

Regarding the volatile compounds, previous studies showed that sterile filtration combined to maceration resulted in a reduction of the fatty acids extracted, but it increased the fatty acid content of the yeast membranes, thus increasing the fermentative capacity without affecting either cell growth or cell viability [61]. In this case, only a cold static sedimentation was applied in both in V1 (prior to inoculation) and in V2 (after maceration and prior to inoculation), which had much smaller effects than sterile filtration on the must composition and the amount of nutrient available to the yeast [62]. However, a partial effect was observed, as the resulting V2 (macerated) wines presented eventually a higher sugar content, slightly less measured alcohol (%) and lower abundances of the main esters of saturated linear fatty acids (mainly ethyl octanoate and decanoate) than V1 wines. V1 wines showed instead higher contents of non-esterified higher alcohols with strong impact on the flavor (e.g., phenylethyl alcohol), which were present mostly as their relative acetate esters in V2 wines. However, the lower efficiency of production of fermentation products could in this case also be associated to the inhibitory effect of the higher phenolic content on the yeast, as reported [21].

Then, the pre-fermentative macerations increased also the content of C6 alcohols extracted in the must, as earlier reported [63]; these compounds have been proposed also as potential markers of varietal authenticity [64], besides influencing the final character of the wines with green fresh vegetable, cut-leaf aromas [55].

Regarding the sensory analysis, the strongest factor identified was a temporal trend associated to the increase in the sourness, saltness, sweetness, warmness, dried fruit, tropical fruit and apple descriptors, together with the overall quality judgment evaluation. Besides, no clear-cut separations between the two wines was observed, although after nine months the non-macerated V1 displayed a higher overall quality and was characterized by apple, dried fruit and tropical fruit aromas, all strongly correlating across PC1 with the overall judgment. Pear and olfactory intensity, together with spicy, sourness and saltness characterized more V2 after nine months in bottle, than V1. Particularly the pear descriptor was indicated as a typical characteristic of Austrian Pinot blanc wines [10,11], but a high value of the pear descriptor is not necessarily associated with the overall quality of Pinot blanc wine [10], as also reported in the present study. The temporal trend was driven mainly by the increase in various higher linear ethyl and higher acetate esters and it was associated accordingly to higher perceived intensity for apple, tropical and dried fruits descriptors, and a higher overall quality judgment. Overall, the two wines displayed an increase in the sensory quality over time assigned by the panel, although the two profiles were different: V1 was characterized by apple, tropical and dried fruits aromas, whereas V2 by spicy, pear aroma, sourness and olfactory intensity. Over time, a consistent decrease in off-odor and fresh vegetative descriptor paralleled the increasing overall quality associated particularly to V1 after nine months.

## 5. Conclusions

In the present work, the effects of two different vinification of Pinot blanc were elucidated observing the overtime evolution of several chemical and sensory parameters. The two wines displayed different profiles after the sixth month after bottling, showing that the control wine evolved faster than the experimental wine. Parallel effects were observed also in the profile of phenolic and volatile compounds. The control wine received a higher overall quality judgement by the trained panel (at three, six and nine months in bottle), being described mostly by tropical fruit and apple aromas. Instead, the sensory descriptors that characterized the experimental wine after nine months of storage were olfactory intensity, spicy and pear aromas. As the main factor was the application of a pre-fermentative cold maceration in the experimental wine, these trends were explained by the higher abundance of polyphenols in the macerated wine, causing a partial yeast stress effect.

As a future perspective, the relationship between sensory and chemical results could be further investigated, to point out the chemical markers that are related to Pinot blanc wines obtained from different geographical areas; indeed, the geographical factor might have an impact, as also appears from comparing previous reports with the present work. This research is crucial to define the sensory identity of the different wine styles obtained from Pinot blanc, both for marketing studies, assessment of their authenticity and to propose winemaking guidelines for this grape variety.

## Figures and Tables

**Table 1:** Procedures applied for the winemaking.

Codes	Operation	Sampling Points	
		V1	V2
**T0 (M)**	Before pressing	X	X
**T1**	Prefermentative cold maceration with pectolytic enzyme		X
**T2**	Two pressing steps at 1 bar	X	X
**T3**	Two pressing steps at 2 bar	X	X
**T4**	Cold sedimentation	X	X
**T5**	At half of fermentation	X	X
**T6**	Addition of yeast autolysate		X
**T7**	End of fermentation	X	X
**T8**	Cold stabilization	X	X
**T9**	Before addition of bentonite	X	X
**T10**	After bentonite clarification		X
**T11**	Pre-filtration	X	X
**T12**	After-filtration	X	X
**T13**	Bottling (**W0**)	X	X
**T14**	3 months into bottle (**W3**)	X	X
**T15**	6 months into bottle (**W6**)	X	X
**T16**	9 months into bottle (**W9**)	X	X

Empty cells correspond to operations that were not performed for the control wine (**V1**) but were performed for the experimental wine (**V2**). Samples were taken at the end of the shown operation. X = operation performed; empty space = operation not performed.

**Table 2:** Compounds identified by HPLC-DAD and HPLC-MS analysis.

	Assignment	R.t. (*min*)	Full MS (*m/z*)	MS/MS (*m/z)*	UV-Vis λ_max_ (*nm*)	Identification
**1**	gallic acid, hexoside ^(*)^	12.4	331 (ESI -)	153 ← 331	267	Assigned by DAD (λ_MAX_) and MS^2^ analysis.
**2**	gallic acid	17.1	169 (ESI -)	*na*	267	Standard injection.
**3**	(-)-gallocatechin	24.0	305 (ESI -)	125, 219 ← 305	279	Fluorescence analysis. Standard injection; assigned by DAD (λ_MAX_) and MS^2^ analysis.
**4**	glutathionylcaftaric acid (GRP)	24.6 ^2^	618 (ESI +)	135, 179 ← 618	297, 327	Assigned by DAD (λ_MAX_) and MS^2^ analysis.
**5**	caftaric acid	26.8 ^1^, 28.0 ^2^	311 (ESI -)	135, 179 ← 311	297, 327	Standard injection (*trans* isomer).
**6**	(-)-epigallocatechin	31.4	305 (ESI -)	125, 219 ← 305	279	Fluorescence analysis. Standard injection; assigned by DAD (λ_MAX_) and MS^2^ analysis.
**7**	(+)-catechin	33.1	289 (ESI -)	*na*	279	Fluorescence analysis. Standard injection.
**8**	coutaric acid	33.8 ^1^, 34.5 ^2^	295 (ESI -)	163 ← 295	295, 308	Standard injection; assigned by DAD (λ_MAX_) and MS^2^ analysis.
**9**	*trans-*caffeic acid	37.0	179 (ESI -)	*na*	297, 327	Standard injection.
**10**	(-)-epicatechin	38.5	289 (ESI -)	*na*	279	Fluorescence analysis. Standard injection.
**11**	*p*-coumaric acid	45.5	163 (ESI -)	*na*	295, 308	Standard injection.
**12**	astilbin	49.8	449 (ESI -)	125, 285, 303 ← 449	290, ~340	Standard injection; assigned by DAD (λ_MAX_) and MS^2^ analysis.
**13**	taxifolin ^(*)^	50.8	303 (ESI -)	125, 285 ← 303	290, ~340	Standard injection; assigned by DAD (λ_MAX_) and MS^2^ analysis.

^1^*cis* isomer; ^2^ *trans* isomer. *na* = not acquired. R.t. error = ± 0.5 min; mass error = ± 0.3 *m/z*; λ_MAX_ error = ± 2 nm. (*) Tentative assignment.

**Table 3:** List of volatile compounds identified by HS-SPME-GC/MS in Pinot blanc.

**WINES**				
**Compound Name**	Base Peak ^(^*^)^ **(*m/z*)**	Retention Time ^(^**^)^ **(min)**	Linear Retention Index ^(^***^)^	**Odor Descriptor**
***Ethyl Esters***				
**ethyl butanoate**	71;43	8.9	1033 (ref.: *DB-Wax, **1035*** [27]; *PEG -{H2 carrier}, **1044*** [28])	Apple ^b^
**ethyl hexanoate**	88	16.6	1232 (ref.: *DB-Wax, **1232*** [29]; *FFAP*, ***1243*** [30])	Apple peel, fruit ^a^
**ethyl octanoate**	88	25.4	1436 (ref.: *DB-Wax, **1433*** [29]; *PEG {H_2_ carrier}, **1436*** [28])	Fruit, fat ^a^
**ethyl nonanoate**	88	29.6	1535 (ref.: *Supelcowax-10,* ***1537****,* [31])	Waxy ^c^
**ethyl decanoate**	88	33.8	1638 (ref.: *SP-1000, **1644*** [32]; *Innovax, **1630**,* [33])	Grape ^a^
**diethyl succinate**	101	35.2	1674 (ref.: *DB-Wax, **1687*** [33])	Wine, fruit ^b^
**ethyl 9-decenoate**	88;55	35.8	1689 (ref.: *DB-Wax, **1688*** [34])	Fruity ^b^
**ethyl dodecanoate**	88	41.4	1841 (ref.: *DB-Wax, **1856*** [35])	Leaf ^b^
**ethyl tetradecanoate**	88	48.2	2050 (ref.: *DB-Wax, **2070*** [35])	Ether ^b^
***Acetate esters***				
**ethyl acetate**	43	5.2	795 (ref.: *DB-Wax, **890*** [36])	Pineapple, nail polish ^a^
**isoamyl acetate**	43	11.9	1119 (ref.: *DB-Wax, **1126*** [37])	Banana ^a^
**hexyl acetate**	43	18.3	1271 (ref.: *DB-Wax, **1279*** [35])	Fruit, herb ^b^
**octyl acetate**	43	27.1	1474 (ref.: *DB-Wax, **1490*** [35])	Fruit ^b^
**phenylethyl acetate**	104	40.4	1812 (ref.: *ZB-Wax, **1811*** [38])	Rose, honey, tobacco ^a^
***Other Esters***				
**methyl octanoate**	*74*	23.4	1388 (ref.: *Innowax, **1386*** [39]; ZB-*Wax, **1386*** [38])	Orange ^b^
**isopentyl hexanoate**	70	26.4	1458 (ref.: *DB-Wax, **1469*** [35]; *BP-20, **1450*** [40])	Fruity ^c^
**isobutyl octanoate**	57	30.3	1551 (ref.: *ZB-Wax, **1550*** [38])	Fruity ^b^
**methyl decanoate**	74	32.0	1593 (ref.: *HP-Wax, **1593*** [41]; *ZB-Wax, **1586*** [38])	Wine ^b^
**isoamyl octanoate**	70	34.5	1657 (ref.: *ZB-Wax, **1658*** [38]; *DB-FFAP,* ***1647*** [42])	Fruity **^c^**
**isopropyl dodecanoate**	43	41.1	1831 (ref.: *ZB-Wax, **1832*** [43])	Green ^c^
**isoamyl decanoate**	70	42.1	1860 (ref.: *ZB-Wax, **1859*** [38])	Waxy ^c^
**ethyl isoamyl succinate**	101	43.5	1898 (ref.: *DB-Wax, **1907*** [34])	(not found)
***Acids***				
**acetic acid**	43	26.0	1448 (ref.: *DB-Wax, **1439*** [44])	Sour, pungent, vinegar ^a^
**octanoic acid**	60	48.4	2057 (ref.: *DB-Wax, **2050*** [45])	Sweet, Cheese ^a^
**nonanoic acid**	60	50.7	2161 (ref.: *DB-Wax, **2159*** [46])	Green, Fat ^b^
***C_6_ Alcohols***				
***n*-hexanol**	56	21.8	1352 (ref.: *DB-Wax, **1360*** [47])	Resin, flower, green ^a^
***Higher Alcohols***				
**isobutyl alcohol**	43	11.0	1094 (ref.: *DB-Wax, **1093*** [35])	Ethereal, nail polish ^c^
**isoamyl alcohol**	55	15.5	1207 (ref.: *HP-Innowax, **1206*** [48])	Whiskey, malt, burnt ^a^
**2,3-butanediol**	45	29.7	1573 (ref.: *DB-Wax, **1580*** [35])	Fruit, onion ^b^
***n*-octanol**	41	30.5	1556 (ref.: *DB-Wax, **1561*** [49])	Chemical, metal, burnt ^b^
***n*-decanol**	70;55	38.4	1759 (ref.: *HP-Innowax, **1764*** [50])	Fat ^b^
**phenylethyl alcohol**	91	43.7	1905 (ref.: *Innowax, **1905*** [33])	Honey, spicy, rose, lilac ^a^
***Terpenes***				
**citronellol**	69;41	38.5	1762 (ref.: *BP-20, **1762*** [51])	Rose ^b^
***Norisoprenoids***				
**β-damascenone**	69	40.6	1819 (ref.: *DB-Wax, **1814*** [29])	Rose, honey, plum ^a^
**MUSTS**				
**Compound Name**	Base Peak ^(^*^)^ **(*m/z*)**	Retention Time ^(^**^)^ **(min)**	Linear Retention Index ^(^***^)^	**Odor Descriptor**
***Ethyl Esters***				
**ethyl butanoate**	71;43	8.9	1033 (ref.: *DB-Wax, **1035*** [27]; *PEG -{H2 carrier}, **1044*** [28])	Apple ^b^
**ethyl octanoate**	88	25.4	1436 (ref.: *DB-Wax, **1433*** [29]; *PEG {H_2_ carrier}, **1436*** [28])	Fruit, fat ^a^
**ethyl decanoate**	88	33.8	1638 (ref.: *SP-1000, **1644*** [32]; *Innovax, **1630*** [33])	Grape ^a^
**diethyl succinate**	101	35.2	1674 (ref.: *DB-Wax, **1687*** [33])	Wine, fruit ^b^
**ethyl dodecanoate**	88	41.4	1841 (ref.: *DB-Wax, **1856*** [35])	Leaf ^b^
***C_6_ Compounds***				
**hexanal**	44	10.5	1079 (ref.: *DB-Wax, **1077*** [27])	Grass, tallow, fat ^b^
**2-(*E*)-hexenal**	41	15.9	1216 (ref.: *DB-Wax, **1215*** [52])	Green, leaf ^b^
***n*-hexanol**	56	21.8	1352 (ref.: *DB-Wax, **1360*** [47])	Resin, flower, green ^b^
**2-hexen-1-ol**	57	24.1	1405 (ref.: *HP-Innowax, **1408*** [53])	Green, leaf, walnut ^b^
***Other Esters***				
**isoamyl octanoate**	70	34.5	1657 (ref.: *ZB-Wax, **1658*** [38])	Fruity ^c^
**isopropyl dodecanoate**	43	41.1	1831 (ref.: *ZB-Wax, **1832*** [41])	Green ^c^
***Higher Alcohols***				
**phenylethyl alcohol**	91	43.7	1905 (ref.: *Innowax, **1905*** [33])	Honey, spicy, rose, lilac ^a^

Sensory descriptors were reported after comparison of the literature and several sources available on line: **^a^** Francis and Newton, 2005 [54]; **^b^** Flavornet by Terry Acree and Heinrich Arn (http://www.flavornet.org); **^c^** The Good Scents Company Information System (http://www.thegoodscentscompany.com). ^(^*^)^ When the MS base peak observed did not match the compounds assigned by comparison with the NIST 2011 database, both are indicated. ^(^**^)^ Retention times were approximated to one-digit value. Measurement errors: retention times = ± 0.1 min; mass = ± 0.7 Da. ^(^***^)^ Library references are indicated in brackets with relative LRI for the most similar chromatographic phase and method found (thus, in some cases, more than one reference is indicated).

## Supplementary Materials

The following materials are available online at https://www.mdpi.com/2304-8158/9/4/499/s1, Table S1: Phenolic compounds determined using a calibration curve with HPLC-DAD/FLD; Table S2: Sensory descriptors listed in the evaluation sheet and their definition; Figure S1: V1.W3.A: HPLC-DAD (280 nm) trace and HPLC-FLD trace; Figure S2: Traces of selected phenolic compounds for V1 and V2; Table S3: Complete enological parameters dataset; Table S4: Two-way ANOVA for *trans*-caftaric acid, GRP, astilbin and taxifolin in relation to vinification and time factors; Figure S3: PCA of HPLC-FLD peaks; Figure S4: Chromatogram examples of V1.W6 and V2.W6 by GC-MS; Table S5: Two-way ANOVA of GCMS variables in relation to vinification and time; Table S6: Two-way ANOVA of the sensory data in relation to vinification and time.
