# Supplementary material for: Pinot Blanc: Impact of the Winemaking Variables on the Evolution of the Phenolic, Volatile and Sensory Profiles
Source: Foods. 2020 Apr 15;9(4):499. doi: 10.3390/foods9040499 (PMC7230968; doi:10.3390/foods9040499)
Supplement: Supplementary file 1 [file foods-09-00499-s001.pdf]

Supplementary material

Pinot blanc: effects of the winemaking variables on the evolution of the phenolic, volatile and sensory profiles

Amanda Dupas de Matos [1,2], Edoardo Longo [1,3,*] Danila Chiotti [4], Ulrich Pedri [4], Daniela Eisenstecken [5], Christof Sanoll [5], Peter Robatscher [5] and Emanuele Boselli [1,3]

1 Faculty of Science and Technology, Free University of Bozen-Bolzano, Piazza Università 5, 39100 Bolzano, Italy; emanuele.boselli@unibz.it (E.B.); edoardo.longo@unibz.it (E.L.)
2 FEAST and Riddet Institute, Massey University, Palmerston North 4410, New Zealand; a.dupasdematos@massey.ac.nz (A.D.M)
3 Oenolab, NOI Techpark, via Alessandro Volta 13, 39100 Bolzano BZ, Italy.
4 Institute for Fruit Growing and Viticulture, Laimburg Research Center, Laimburg 6, I-39051 Pfatten, Italy; danila.chiotti@laimburg.it (D.C.); ulrich.pedri@laimburg.it (U.P.)
5 Institute for Agricultural Chemistry and Food Quality, Laimburg Research Center, Laimburg 6, I-39051 Pfatten, Italy; daniela.eisenstecken@laimburg.it (D.E.); christof.sanoll@laimburg.it (C.S.); peter.robatscher@laimburg.it (P.R.)
* Correspondence: edoardo.longo@unibz.it; Tel.: +39 0471 017691

Received: 17 March 2020; Accepted: 13 April 2020; Published: 14 April 2020

SUPPLEMENTARY MATERIAL

Table S1. Phenolic compounds determined using a calibration curve with HPLC-DAD/FLD.

DAD					
Name	Rt (min)	Slope (mAU µM^{-1})	R^2	Range calibrated (µM)	Average RSD%
**gallic acid**	17.1	3225	> 0.999	3 – 53	< 2%
**protocatechuic acid**	25.0	1648	> 0.999	3 – 51	< 2%
**vanillic acid**	36.5	2026	> 0.999	3 - 51	< 2%
**syrigic acid**	37.9	4155	> 0.999	3 - 53	< 2%
***p*-hydroxybenzoic acid**	32.6	1262	> 0.999	3 - 58	< 2%
**chlorogenic acid**	32.5	5408	> 0.999	2 - 50	< 2%
**(+)-catechin (DAD)**	33.1	1416	> 0.999	2 - 50	< 2%
**caffeic acid**	37.0	4221	> 0.999	3 - 52	< 2%
**(-)-epicatechin (DAD)**	38.5	1486	> 0.999	3 - 58	< 2%
***p*-coumaric acid**	45.5	5830	> 0.999	3 - 53	< 2%
**ferulic acid**	48.9	4239	> 0.999	2 - 51	< 2%
**FLD**					
Name	Rt: (min)	Slope (mV µM^{-1})	R^2	Range calibrated (µM)	Average RSD%
**(+)-catechin (FLD)**	33.1	369705	> 0.999	2 - 50	< 2%
**(-)-epicatechin (FLD)**	38.5	403944	> 0.999	3 - 58	< 2%

Table S2. Sensory descriptors listed in the evaluation sheet and their definition.

Sensory descriptor	Definition
Visual Evaluation	
Clarity	Absence of veiling or suspension in the wine
Color intensity	Intensity of yellow color
Olfactory Evaluation	
Overall intensity	Total smell intensity perceived through the nose
Floral	Rose, elder aromas
Tree Fruit (apple)	Apple aroma
Tree Fruit (pear)	Pear aroma
Tropical fruit	Banana, pineapple, mango aromas
Dried fruit	Raisin, dried apricot, dried plum aromas
Spicy	Licorice, black pepper aromas
Fresh vegetative	Sage, mint aromas
Cleanness	Absence of faults/taints/unpleasant odors
Off-odor	Presence of faults/taints/unpleasant odors
Gustatory evaluation	
Warmness	Sensation of alcohol (warm)
Sweetness	Taste of sucrose solution
Sourness	Taste of tartaric acid solution
Saltiness	Taste of sodium chloride solution
Bitterness	Taste of caffeine solution
Astringency	Tactile sensation related to drying of the mouth, sensation of alum solution
**Overall judgement**	Objective evaluation on the overall quality of wine

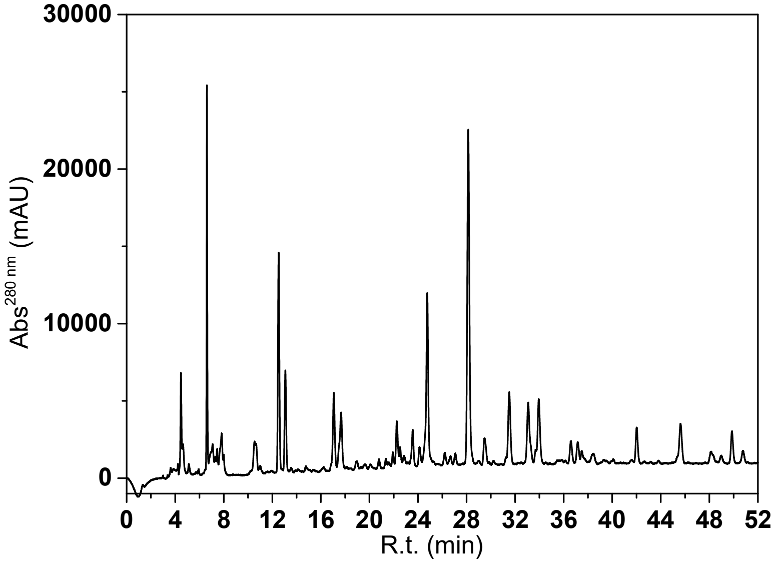

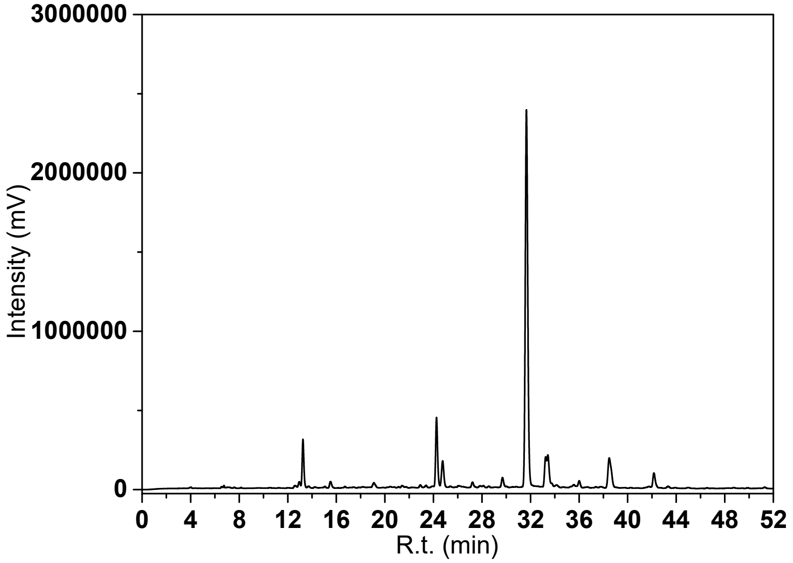

Figure S1. V1.W3.A. **Left**: HPLC-DAD (280 nm) trace. **Right**: HPLC-FLD trace (λexc. = 276 nm; λem. = 316 nm).

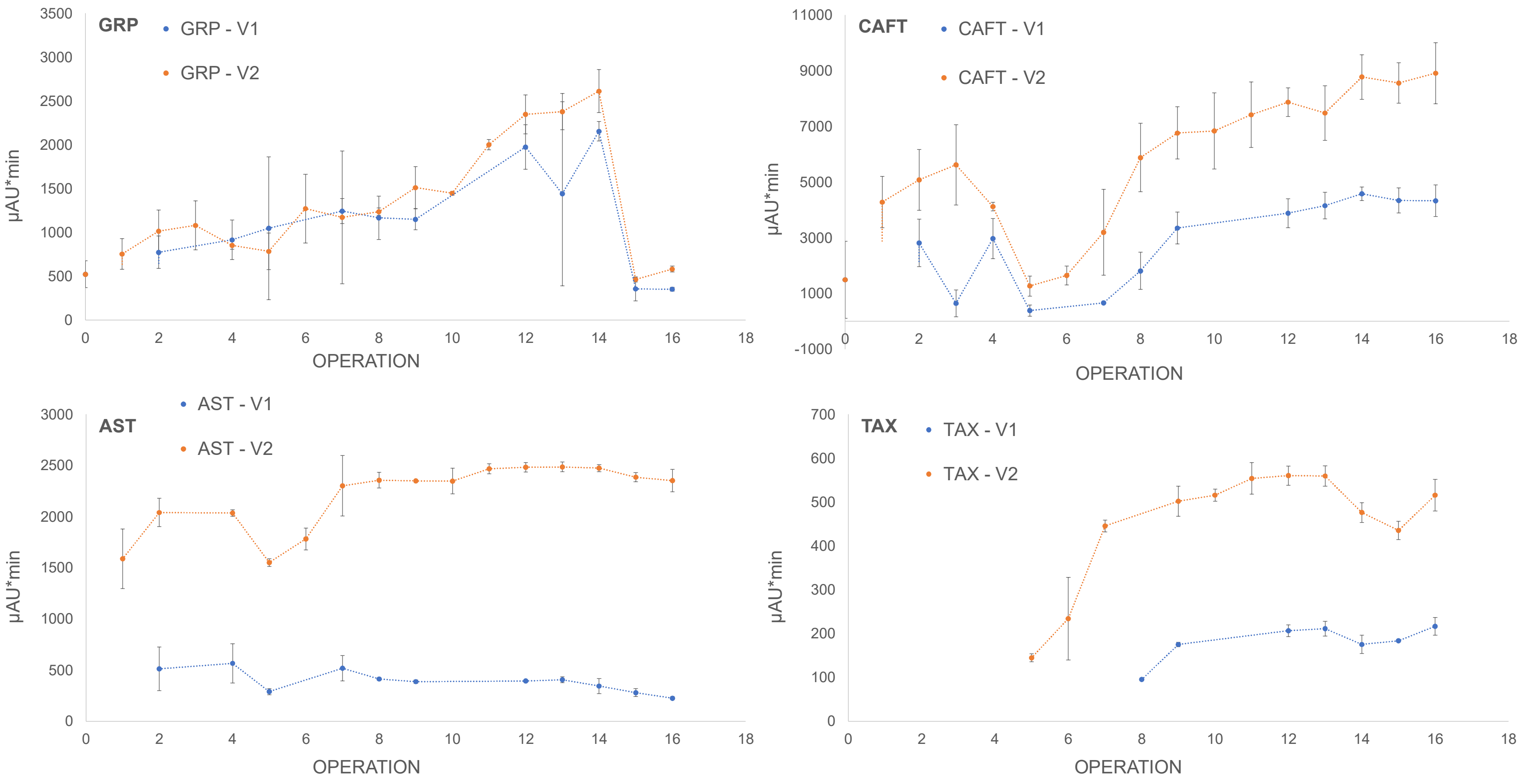

Figure S2. Traces of selected phenolic compounds for **V1** (blue) and **V2** (red). GRP: *S*-glutathionylcaftaric acid; CAFT: *trans*-caftaric acid; AST: astilbin; TAX= taxifolin. The numbers reported in the X axis corresponds to the sample points reported in Table 1 (e.g., 4 and 8 correspond to T4 and T8, respectively).

DATE	TIME	SAMPLE	sugars (g·L^{-1})	° babo	pH	total ac. (g·L^{-1})	malic ac. (g·L^{-1})	tartaric ac. (g·L^{-1})	potassium (g·L^{-1})	AA (mg·L^{-1})	NH$_4$ (mg·L^{-1})	YAN (mg·L^{-1})	gluconic ac. (g·L^{-1})
17/09/2018	T0	MUST	254.0	20.08	3.41	6.36	2.07	7.02	1.583	102	41	145	0.54

DATE	TIME	SAMPLE	sugars	babo	pH	total ac.	malic ac.	tartaric ac.	potassium	AA	NH$_4$	YAN	gluconic ac.	polyphenols (mg·L^{-1})
18/09/2018	T4	V1.1	251.6	20.26	3.5	5.02	2.38	4.85	1.448	117	39	158	1.05	405
		V1.2	253.4	20.19	3.48	5.03	2.4	4.95	1.507	120	42	163	0.75	379
		V1.3	258.2	20.27	3.47	5.09	2.45	4.98	1.426	122	38	160	0.61	379
		V2.1	254.5	20.15	3.5	4.74	2.16	4.26	1.345	131	38	170	1.24	411
		V2.2	254.4	20.24	3.51	4.73	2.32	4.42	1.424	142	38	177	0.75	436
		V2.3	257.9	20.21	3.51	4.72	2.18	4.38	1.305	140	35	175	1.13	435

DATE	TIME	SAMPLE	alcohol	extract	sugars	gf	pH	total ac.	volatile ac. (g·L^{-1})	malic ac.	tartaric ac.	lactic ac.	potassium
02/10/2019	T7	V1.1	14.95	23.95	1.34		3.49	6.44	0.26	1.29	3.35	0.22	0.766
		V1.2	14.97	23.28	1.43		3.5	6.18	0.27	1.24	3.47	0.26	0.746
		V1.3	14.99	23.45	1.31		3.5	6.24	0.26	1.3	3.39	0.28	0.782
03/10/2019		V2.1	14.37	27.46	2.85	1.42	3.46	7.34	0.2	1.83	2.65	0.41	0.818
		V2.2	14.43	26.98	2.85	1.45	3.5	6.84	0.23	1.63	2.56	0.31	0.837
		V2.3	14.37	27.61	3.17	1.75	3.49	7.03	0.22	1.76	2.6	0.33	0.794

DATE	TIME	SAMPLE	alcohol	extract	sugars	gf	pH	total ac.
05/10/2019	END OF **V2** FERMENTATION (2 days AFTER **V1**)	V2.1	14.57	27.33	2.62	1.01	3.48	7.25
		V2.2	14.66	26.45	2.24	0.67	3.52	6.82
		V2.3	14.59	27.01	2.2	0.64	3.52	6.97

			alcohol	extract	sugars	pH	total ac.	volatile ac.	malic ac.	lactic ac. ($g \cdot L^{-1}$)	free SO_2 ($mg \cdot L^{-1}$)	total SO_2 ($mg \cdot L^{-1}$)
31/01/2019	T10	V1.1	14.96	22.38	2.18	3.43	5.7	0.3	1.29	0.33	18.6	71
		V1.2	14.94	21.28	1.82	3.43	5.44	0.31	1.15	0.18	20.7	75
		V1.3	14.94	21.54	1.92	3.43	5.52	0.31	1.16	0.3	17.1	71
		V2.1	14.44	25.7	2.59	3.45	6.73	0.22	1.78	0.32	15.4	62
		V2.2	14.57	24.76	2.42	3.48	6.21	0.25	1.64	0.34	16.7	71
		V2.3	14.46	25.75	2.48	3.49	6.48	0.23	1.68	0.41	17	70

			free SO_2	total SO_2
26/02/2019	W0 - before SO2	V1.1	19	74
W0 before SO2 add.		V1.2	22	78
		V1.3	19	75
		V2.1	16	67
		V2.2	19	76
		V2.3	17	69

			free SO_2	total SO_2
26/02/2019	W0 - after SO2	1.1	27.7	88
W0 after SO2 add.		1.2	26	83
		1.3	20.4	75
		2.1	22.4	80
		2.2	24.8	88
		2.3	17.9	71

			alcohol	extract	sugars ($g \cdot L^{-1}$)	pH	total ac.	volatile ac.	malic ac.	lactic ac.	free SO_2	total SO_2
07/06/2019	W3	V1.1	14.96	21.32	1.36	3.43	5.58	0.31	1.26	0.38	22.8	77
W3		V1.2	14.97	20.58	1.13	3.45	5.29	0.31	1.3	0.5	26.4	81
		V1.3	14.93	21.3	1.18	3.44	5.38	0.31	1.29	0.64	28.5	89
		V2.1	14.45	25.02	2.17	3.47	6.49	0.22	1.9	0.57	27.2	89
		V2.2	14.55	24.59	1.91	3.49	6.08	0.24	1.73	0.52	29.5	96
		V2.3	14.49	24.73	2.05	3.49	6.16	0.24	1.79	0.59	29.2	94

			alcohol	extract	sugars	pH	total ac.	volatile ac.	malic ac.	lactic ac.	free SO_2	total SO_2
11/09/2019	W6	V1.1	15.12	20.98	1.72	3.43	5.42	0.31	1.33	0.45	21.9	77
W6		V1.2	15.1	20.77	1.27	3.45	5.25	0.32	1.2	0.42	24.2	80
		V1.3	15.03	22.05	1.33	3.44	5.54	0.34	1.21	0.64	29.3	92
		V2.1	14.56	25.48	2.05	3.47	6.51	0.24	1.8	0.57	25.5	89
		V2.2	14.64	24.45	2	3.5	6.03	0.25	1.61	0.47	29.8	100
		V2.3	14.61	25.11	2.33	3.48	6.2	0.26	1.75	0.54	27.2	93

Table S3. Complete enological parameters dataset.

Table S4. Two-way ANOVA for *trans*-caftaric acid (CAFT), GRP (*S*-glutathionylcaftaric acid), astilbin (AST) and taxifolin (TAX) in relation to vinification and time factors. Selected α = 0.05.

**GRP**	Sum of sqrs	df	Mean square	F	**p (same)**
VINIF:	774370	1	774370	4.728	**0.04609**
TIME:	1.73E+07	3	5.76E+06	35.14	**< 0.0001**
Interaction:	612116	3	204039	1.246	0.3282
Within:	2.46E+06	15	163792		
Total:	2.14E+07	22			

**CAFT**	Sum of sqrs	df	Mean square	F	**p (same)**
VINIF:	9.98E+07	1	9.98E+07	193.8	**< 0.0001**
TIME:	2.80E+06	3	934267	1.813	0.1854
Interaction:	1.28E+06	3	425059	0.8249	0.4992
Within:	8.24E+06	16	515295		
Total:	1.12E+08	23			

**AST**	Sum of sqrs	df	Mean square	F	**p (same)**
VINIF:	2.67E+07	1	2.67E+07	8407	**< 0.0001**
TIME:	91977.5	3	30659.2	9.637	**< 0.0001**
Interaction:	2463.25	3	821.082	0.2581	0.8545
Within:	50901.5	16	3181.34		
Total:	2.69E+07	23			

**TAX**	Sum of sqrs	df	Mean square	F	**p (same)**
VINIF:	540177	1	540177	1106	**< 0.0001**
TIME:	22156.3	3	7385.44	15.12	**< 0.0001**
Interaction:	6995.23	3	2331.74	4.772	**0.01462**
Within:	7817.43	16	488.589		
Total:	577146	23			

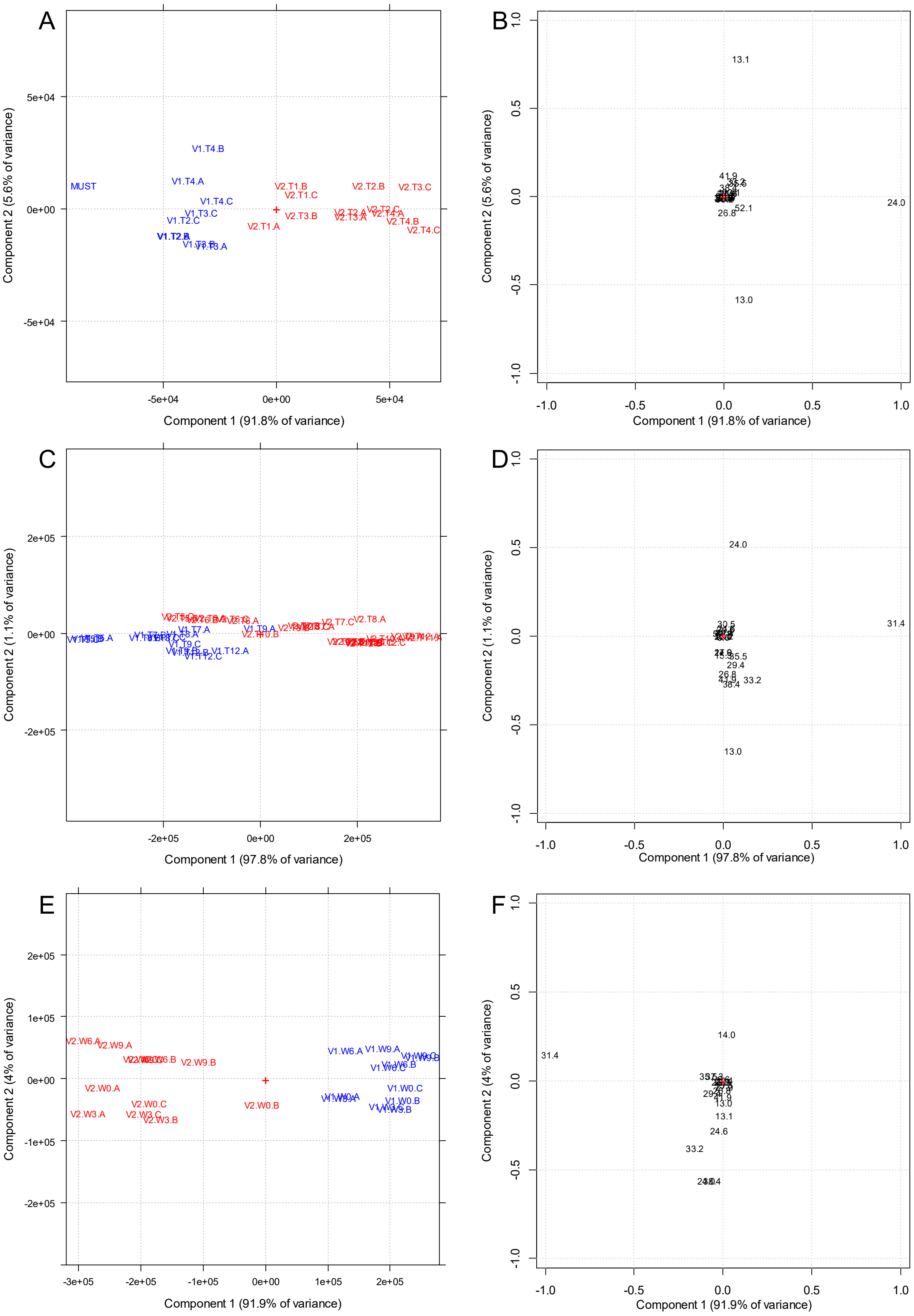
A
Component 2 (5.6% of variance)
Component 1 (91.8% of variance)
MUST
V1.T4.B
V1.T4.A
V1.T4.C
V1.T3.C
V1.T2.C
V2.T1.B
V2.T1.C
V2.T1.A
V2.T3.B
V2.T2.B
V2.T3.C
V2.T4.B
V2.T4.C
B
13.1
24.0
52.1
26.8
13.0
C
Component 2 (1.1% of variance)
Component 1 (97.8% of variance)
V1.T12.A
V2.T7.C
V2.T8.A
D
24.0
31.4
29.4
33.2
13.0
E
Component 2 (4% of variance)
Component 1 (91.9% of variance)
V2.W6.A
V2.W9.A
V2.W9.B
V2.W0.A
V2.W0.C
V2.W3.A
V2.W3.B
V2.W0.B
V1.W6.A
V1.W9.A
V1.W0.C
V1.W0.B
F
31.4
14.0
13.1
24.6
33.2

Figure S3. PCA of HPLC-FLD peaks. A) PC1 *vs* PC2 score plot for must samples; B) PC1 *vs* PC2 loading plot for must samples; C) PC1 *vs* PC2 score plot for fermentation and stabilization samples; D) PC1 *vs* PC2 loading plot for fermentation and stabilization samples; E) C) PC1 *vs* PC2 score plot for bottled samples; F) PC1 *vs* PC2 loading plot for bottled samples. Loadings are indicated with their retention times as labels. Blue font indicates **V1** samples; red font indicates **V2** samples. Retention time labels were corrected for the shift from DAD retention times (0.3 min) using the peaks of (+)-catechin and (-)-epicatechin as reference. "A, B, C" in the score plots' labels indicate the three replicates.

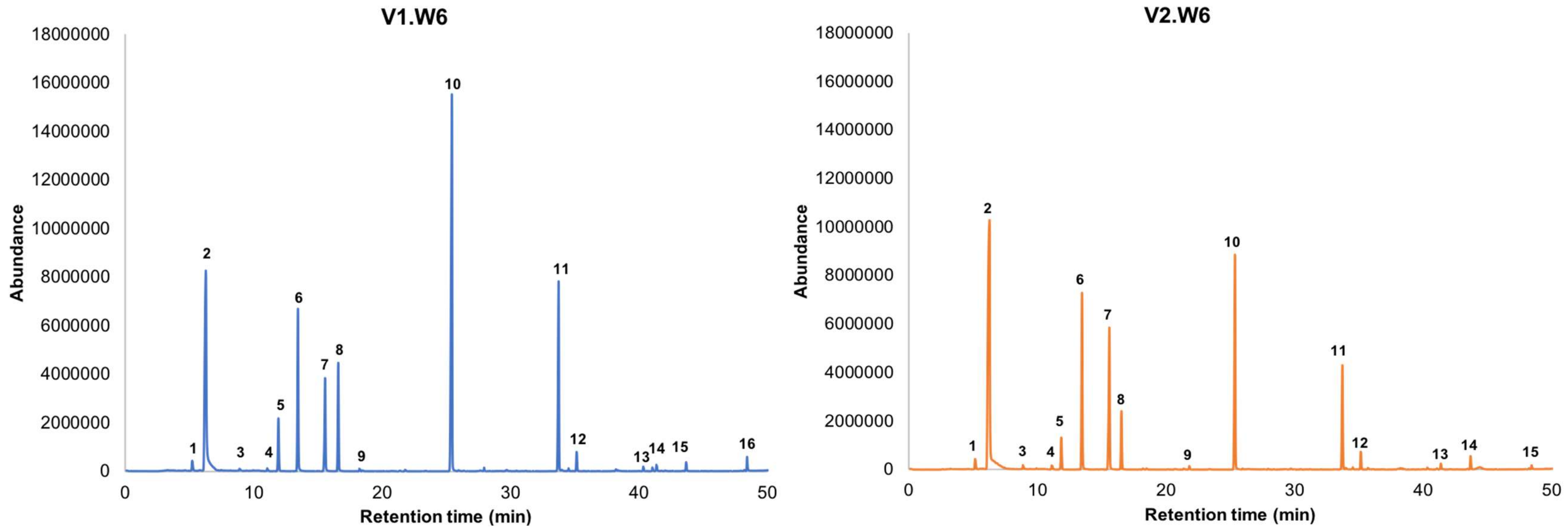

Figure S4. Left: Chromatogram examples of V1.W6 and V2.W6 by GC-MS. 1) ethyl acetate (Rt. 5.20 min); 2) ethanol (Rt. 6.20 min); 3) ethyl butanoate (Rt. 8.92 min); 4) isobutyl alcohol (Rt. 10.98 min); 5) isoamyl acetate (Rt. 11.95 min); 6) **I.S. 2-methyl-3-pentanol** (Rt. 13.41 min); 7) isoamyl alcohol (Rt. 15.53 min); 8) ethyl hexanoate (Rt. 16.61 min); 9) hexyl acetate (Rt. 18.29 min); 10) ethyl octanoate (Rt. 25.45 min); 11) ethyl decanoate (Rt. 33.79 min); 12) diethyl succinate (Rt. 35.19 min); 13) phenylethyl acetate (Rt. 40.39 min); 14) ethyl dodecanoate (Rt. 41.42 min); 15) phenylethyl alcohol (Rt. 43.72 min); 16) octanoic acid (Rt. 48.45 min). **Right:** Chromatogram of V2.W6 by GC-MS. 1) ethyl acetate (Rt. 5.20 min); 2) ethanol (Rt. 6.20 min); 3) ethyl butanoate (Rt. 8.92 min); 4) isobutyl alcohol (Rt. 10.98 min); 5) isoamyl acetate (Rt. 11.95 min); 6) **I.S. 2-methyl-3-pentanol** (Rt. 13.41 min); 7) isoamyl alcohol (Rt. 15.53 min); 8) ethyl hexanoate (Rt. 16.61 min); 9) *n*-hexanol (Rt. 21.84 min); 10) ethyl octanoate (Rt. 25.45 min); 11) ethyl decanoate (Rt. 33.79 min); 12) diethyl succinate (Rt. 35.19 min); 13) ethyl dodecanoate (Rt. 41.42 min); 14) phenylethyl alcohol (Rt. 43.72 min); 15) octanoic acid (Rt. 48.45 min).

Table S5. Two-way ANOVA of GCMS variables in relation to vinification and time (α = 0.05). Groups were evaluated by Tukey's *post-hoc* test. *n.i.*: not identified compound.

(I) Table S5.A – VINIFICATION FACTOR

VINIFICATION	ethyl acetate	ethyl butanoate	isoamyl acetate	isoamyl alcohol	ethyl hexanoate	hexyl acetate	*n*-hexanol	ethyl octanoate	acetic acid	isoamyl hexanoate
V1	a	b	a	b	a	a	b	a	a	a
V2	b	a	b	a	b	b	a	b	b	b
p-value	0.007	< 0.0001	0.009	0.038	< 0.0001	< 0.0001	< 0.0001	0.002	< 0.0001	0.009

VINIFICATION	ethyl nonanoate	2,3-butanediol	ethyl decanoate	3-methylbutyl octanoate	phenylethyl acetate	ethyl dodecanoate	isoamyl decanoate	phenylethyl alcohol	ethyl tetradecanoate	octanoic acid
V1	a	a	a	a	a	a	a	b	a	a
V2	b	b	b	b	b	b	b	a	b	b
p-value	0.002	0.002	0.003	0.005	< 0.0001	0.000	< 0.0001	< 0.0001	0.001	0.001

(II) Table S5.B – TIME FACTOR

MONTH	ethyl acetate	ethyl butanoate	10.98 (*n.i.*)	isoamyl acetate	isoamyl alcohol	ethyl hexanoate	hexyl acetate	*n*-hexanol
0	b	c	ab	c	c	c	b	c
3	b	c	ab	c	c	c	b	c
6	b	b	b	b	b	b	ab	b
9	a	a	a	a	a	a	a	a
p-value	< 0.0001	< 0.0001	0.007	< 0.0001	< 0.0001	< 0.0001	0.004	< 0.0001

MONTH	ethyl octanoate	acetic acid	isoamyl hexanoate	ethyl nonanoate	2,3-butanediol	*n*-octanol	ethyl decanoate	isoamyl octanoate
0	b	c	b	b	b	b	b	ab
3	b	bc	b	b	b	b	b	b
6	b	b	b	ab	b	b	b	b
9	b	a	a	a	a	a		a
p-value	< 0.0001	< 0.0001	< 0.0001	0.004	< 0.0001	< 0.0001	0	0.026

MONTH	diethyl succinate	*n*-decanol	isopropyl dodecanoate	ethyl dodecanoate	ethyl isoamyl succinate	phenylethyl alcohol	ethyl tetradecanoate	octanoic acid
0	c	b	b	ab	c	b	bc	b
3	c	b	b	ab	c	ab	c	b
6	b	a	a	b	b	b	ab	b
9	a	a	a	a	a	a	a	a
p-value	< 0.0001	< 0.0001	< 0.0001	0.032	< 0.0001	0.004	0	0.009

(III) Table S5.C - INTERACTION

VINIFICATION*MONTH	ethyl acetate	ethyl butanoate	ethyl hexanoate	21.84 *(n.i.)*	acetic acid	isoamyl hexanoate	ethyl nonanoate	2,3-butanediol
V1*9	a	b	a	b	a	a	a	a
V2*9	b	a	b	a	b	b	b	b
V1*6	c	cd	b	c	c	b	b	bc
V2*6	c	c	c	b	cd	b	b	bc
V1*3	c	e	cd	b	cd	b	b	bc
V1*0	c	e	cde	b	cd	b	b	c
V2*0	c	e	e	b	d	b	b	c
V2*3	c	e	de	b	cd	b	b	c
p-value	0.00017862	0.00252091	< 0.0001	0.042672887	< 0.0001	0.02818427	0.006986364	0.018341138

Table S6. Two-way ANOVA of the sensory data in relation to vinification and time ($\alpha = 0.05$). Groups were evaluated by Tukey's *post-hoc* test.

Table S6.A – VINIFICATION FACTOR

VINIFICATION	Floral	Tropical fruit	Cleanness
V1	a	a	a
V2	b	b	b
p-value	0.000	0.004	0.040

Table S6.B – TIME FACTOR

MONTH	Floral	Tropical fruit	Dried fruit	Fresh vegetative	Warmness	Sweetness	Sourness	Saltness	Overall judgement
3	b	b	b	a	b	b	b	b	b
6	a	b	ab	b	ab	b	b	b	ab
9	a	a	a	b	a	a	a	a	a
p-value	< 0.0001	0.001	0.008	0.012	0.009	0.001	0.003	< 0.0001	0.011

Table S6.C – INTERACTION

INTERACTION	Floral
V1*9	a
V2*9	cd
V1*6	ab
V1*3	cd
V2*6	bc
V2*3	d
p-value	0.006
